# Glofitamab-Associated Immune Effector Cell-Associated Neurotoxicity Syndrome (ICANS) Presenting as Serial Seizures and Responding Positively to Antiseizure Drugs and Anakinra: A Case Report

**DOI:** 10.7759/cureus.60833

**Published:** 2024-05-22

**Authors:** Josef Finsterer

**Affiliations:** 1 Neurology, Neurology and Neurophysiology Center, Vienna, AUT

**Keywords:** meningeal carcinomatosis, cerebral lymphoma, seizures, anakinra, icans

## Abstract

Immune effector cell-associated neurotoxicity syndrome (ICANS) is a well-known side effect of chimeric antigen receptor (CAR) T-cell therapy but has occasionally been described with immune checkpoint inhibitors as well. Glofitamab-associated ICANS with a bispecific monoclonal antibody has rarely been reported.

The patient is a 63-year-old male with a history of mantle cell lymphoma, diagnosed at age 37, and aggressive large-cell B-cell lymphoma, diagnosed at age 50. Despite adequate chemotherapy, immunotherapy, autologous stem cell transplantation, and CAR T-cell therapy, there were several relapses, including meningeal carcinomatosis at age 61 and intracerebral lymphoma at age 62. For this reason, glofitamab was started. One week after the ninth cycle, the patient developed drowsiness, behavioral changes, word-finding difficulties, aphasia, focal to bilateral tonic-clonic seizures, and focal onset seizures, which resolved after 16 days with levetiracetam, valproic acid, lorazepam, and midazolam. Since there was no infectious disease, electrolyte disturbance, metabolic disorder, cardiovascular disease, or relapse of lymphoma, glofitamab-associated ICANS was suspected, and anakinra was administered.

The case shows that ICANS with drowsiness, behavioral changes, aphasia, and seizures can develop with glofitamab and that patients with structural brain abnormalities may be prone to this.

## Introduction

Immune effector cell-associated neurotoxicity syndrome (ICANS) is a newly described side effect that typically occurs after the use of chimeric antigen receptor (CAR) T-cell therapy in 20%-60% of cases [1], but is rarely also caused by immune checkpoint inhibitors (e.g., monoclonal antibodies (CD20 blockers)) [2]. The severity of ICANS varies significantly between mild manifestations and persistent, fatal cerebral edema [1]. The most common clinical manifestations include myoclonus, tremor, headache, hallucinations, writing disorder, ataxia, aphasia, dysarthria, psychosyndrome, cognitive impairment, agitation, delirium, impaired alertness, sensorimotor deficits, incontinence, seizures, multifocal demyelinating leukoencephalopathy, and cerebral edema [1]. Immune effector cell-associated neurotoxicity syndrome with severe symptoms (>grade 3) develops in 12%-30% of patients who receive CAR T-cell therapy [1].

Immune effector cell-associated neurotoxicity syndrome has also been reported during monoclonal antibody therapy [2]. One of these is glofitamab, a bispecific monoclonal antibody (CD20-directed CD-3 T-cell engager) for the treatment of large B-cell lymphoma [3]. The most common side effects include cytokine release syndrome (CRS), dizziness or lightheadedness, a feeling of constant movement of oneself or the environment, headache, swelling of the face, arms, lower legs, or feet, burning numbness, tingling or painful sensations, chest pain, cough, muscle pain, bone pain, and fatigue [4]. Prior reports of ICANS associated with glofitamab have been rare.

## Case presentation

The patient is a 63-year-old man who was diagnosed with Ann Arbor stage IV A mantle cell non-Hodgkin lymphoma (NHL) diagnosed in 1998 by a lymph node biopsy from the left axilla and the right groin (Table 1). Mantle cell lymphoma was treated with cyclophosphamide, etoposide, and prednisolone (CEOP) (six cycles), stem cell mobilization, autologous stem cell transplantation (ASCT), and rituximab (four cycles).

**Table 1:** A timeline of the patient’s medical history m: month; y: year; CEOP: cyclophosphamide etoposide and prednisolone; ASCT: autologous stem cell transplantation; RTX: rituximab; R/CHOP: rituximab, cyclophosphamide, doxorubicin, vincristine, and prednisone; CNS: central nervous system; m: month; MATRIX: methotrexate, cytarabine, thiotepa, and rituximab; CA: chimeric antigen receptor; ICANS: immune effector cell-associated neurotoxicity syndrome

Date (mm/yyyy)	Diagnosis and event	Treatment
07/1998	Mantle cell lymphoma	CEOP, ASCT, RTX
08/2011	Large-cell B-cell lymphoma	R/CHOP, plerixafor, stem cell apheresis
02/2013	Relapse L5 vertebral body	Kyphoplasty, interlaminar fenestration
05/2013	Relapse L5 vertebral body	Radiotherapy, bendamustine, ASCT
05/2020	Immunodeficiency	Intravenous immunoglobulins
05/2021	Mantle cell lymphoma, left thigh	Resection
06/2022	CNS relapse	MATRIX
01/2023	None	CAR T-cell therapy
08/2023	CNS relapse	Glofitamab
09/2023	None	First cycle glofitamab
02/2023	None	Ninth cycle glofitamab
03/2024	ICANS	Steroids, anti-seizure drugs, anakinra

In August 2011, large-cell B-cell NHL (rich in T-cells and histiocytes) was also diagnosed by a lymph node biopsy from the right groin. This was treated with rituximab, cyclophosphamide, doxorubicin, vincristine, and prednisone (R/CHOP, six cycles) plus Myocet, stem cell mobilization with granulocyte stimulating factor (G-CSF) and plerixafor and stem cell apheresis. Due to a relapse in the L5 vertebral body and surrounding soft tissue, kyphoplasty was performed. Three weeks later, he underwent L4/5 and L5/S1 interlaminar fenestration, micro-discectomy, and debridement.

Since 2020, the patient has been receiving repeated intravenous immunoglobulins (IVIG) to strengthen the immune system.

In May 2021, mantle cell lymphoma (blastoid subtype CD20+, CD5-, cyclin D1+, SOX11+, CD10+, Ki67 100%) with infiltration of the left thigh was diagnosed and resected.

In June 2022, the patient developed gait disturbance, flaccid dysarthria, difficulty finding words, visual hallucinations, and subsequently a coma for two weeks. A workup revealed a relapse of the lymphoma with central nervous system (CNS) involvement (Deauville 5), with pleocytosis of 7333/ml CD5-B-cells (Table 1) but without bone marrow infiltration. This was treated with methotrexate, cytarabine, thiotepa, and rituximab (MATRIX, four cycles). During the first cycle, the patient developed delirium, either as a side effect of MATRIX or possibly related to sepsis with *Staphylococcus epidermidis*, which complicated chemotherapy.

In January 2023, he received CAR T-cell therapy (tisagenlecleucel (tisa-cel)). Despite this treatment, he suffered a relapse of CNS involvement seven months later, manifesting as multiple intra-parenchymatous blastomas bilaterally and mantle cell pleocytosis (Table 2). Clinically, the relapse manifested itself as the very first focal to bilateral tonic-clonic seizure with postictal right-sided hemiplegia, speech disturbance, dizziness, and facial spasms. After recovery, he was diagnosed with right-sided hemiparesis with predominantly upper extremity involvement. After two weeks, the leg weakness disappeared completely, but mild weakness of the right upper extremity has persisted since then. He was administered anti-seizure drugs (ASDs) in the form of levetiracetam (2,000 mg/d), and since September 2023, he has also received glofitamab after pretreatment with obinutuzumab (2000mg) and IVIG. He also received G-CSF (13 cycles). In the ninth cycle of glofitamab, he developed a fever, which is why he was also given tocilizumab. He had tolerated the other eight glofitamab cycles without any major side effects, apart from an occasional mild fever.

**Table 2:** The patient's CSF findings between the ages of 61 and 63 Nd: not done; RL: reference limit; *: lymphoma cells; CSF: cerebrospinal fluid

Parameter	RL	05/2022	07/2022	08/2022	09/2022	10/2022	08/2023	03/2024	03/2024
Cell count	0.4	7333*	28	5	2	1	38	7	14
Total protein	15-45 mg/dl	943	185	90	82	61	nd	41	49
Albumin	10-30 mg/dl	>450	111	51	42	33	nd	17	24
Glucose	40-70 mg/dl	13	61	51	49	50	nd	80	82

**Figure 1:**
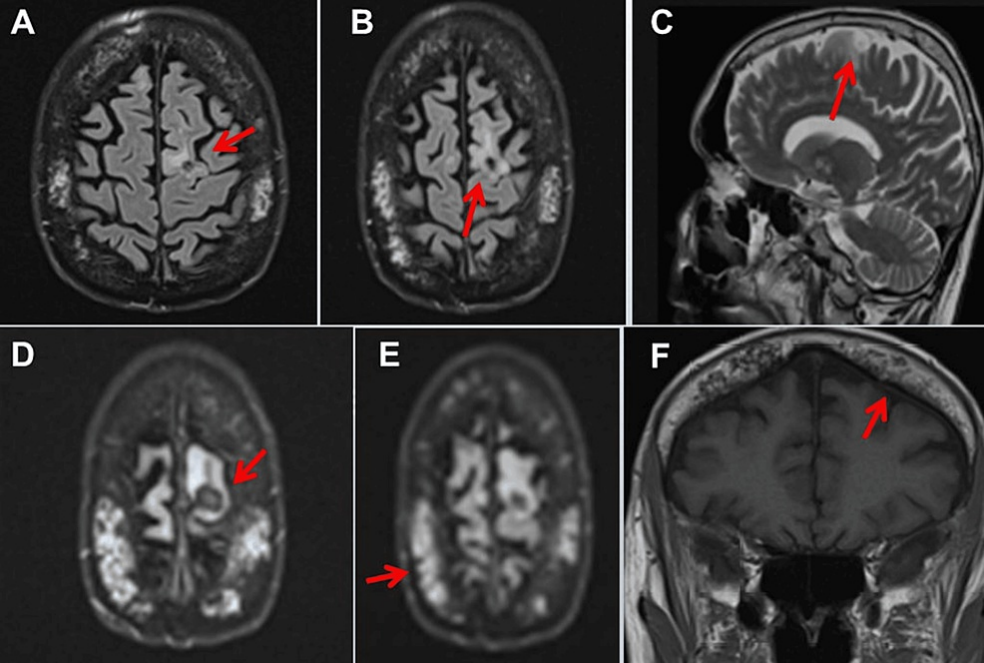
Cerebral MRI at age 63 shows a previously described right frontal mass lesion with perifocal edema and small edematous zones in a left parieto-occipital subcortical, right temporal, and bilateral frontal distribution (panels A to E). There is also evidence for leptomeningeal thickening, possibly secondary to previous meningeal carcinomatosis (panel F).

He was readmitted in March 2024 for visual hallucinations, self-talk, and word-finding problems resulting in Broca’s aphasia, insomnia, drowsiness, and jaw myocloni for approximately 10 seconds. There was no evidence of infectious disease, electrolyte disturbance, metabolic disorder, or lymphoma relapse. The MRI showed the previously described right frontal mass lesion with perifocal edema and small oedematous zones in a left parieto-occipital subcortical, right temporal, and bilateral frontal distribution (Figure 1). The CSF analysis showed no lymphoma cells (Table 2). Dexamethasone was added. On hospital day four, he suffered a focal to bilateral tonic-clonic seizure lasting three minutes. Levetiracetam was increased to 2,500 mg/d. Recurrent focal onset seizures developed over the following days, and on hospital day 11, he suffered another focal to bilateral tonic-clonic seizure lasting four to five minutes. A second lumbar puncture again revealed no lymphoma cells. The frequency of focal-onset seizures, which lasted only a few seconds, further increased to one seizure every five to 10 minutes.

**Figure 2:**
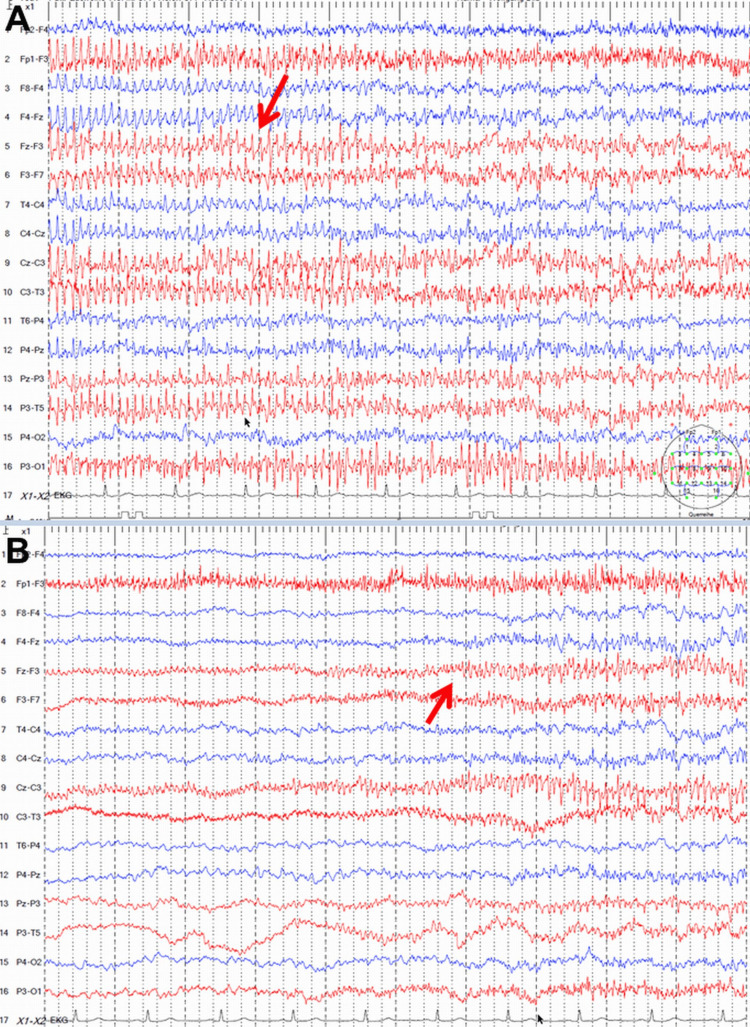
Standard EEG recordings after the third tonic-clonic seizures show electrical seizure activity over the left hemisphere (A), as well as non-specific continuous slowing over the left and intermittent slowing over the right hemisphere (B).

Neurological examination on hospital day 13 revealed Broca’s aphasia, ideomotor apraxia, fluctuations in alertness, recurrent focal onset seizures lasting a few seconds and manifested by right head rotation, repeated gaze deviation to the right, and occasional left arm clonus, intermittent anesthesia to painful stimuli, inconsistent compliance with commands, diffuse weakness of the right upper extremity, and generally decreased tendon reflexes. Levetiracetam was increased to 3,000 mg/d, and lorazepam (4 mg/d) was added. Because focal-onset seizures did not resolve, valproic acid (800 mg/d) was added, as it was the only ASD available that could be additionally administered intravenously. His EEG was abnormal, showing three 50s electrical seizure activity over the left hemisphere, as well as non-specific continuous slowing over the left and intermittent slowing over the right hemisphere (Figure 2). As focal onset seizures persisted, valproic acid was increased to 1,600 mg/d. Since ICANS was suspected, anakinra was also administered on hospital day 15. Because focal-onset seizures continued to occur, midazolam (0.5 mg/h) was added, and a do-not-resuscitate order was issued. Surprisingly, focal-onset seizures decreased from hospital day 20 onwards, and the seizures subsequently disappeared completely and have not recurred since. Postictually, the patient reported amnesia between hospital days 12 and 19. On hospital day 55, the patient was discharged with treatment of levetiracetam and valproic acid.

## Discussion

The presented patient is of interest to ICANS after repeated administration of glofitamab for a relapse of an aggressive B-cell NHL that was diagnosed 13 years previously. Immune effector cell-associated neurotoxicity syndrome was diagnosed as grade 3 according to the American Society for Transplantation and Cellular Therapy (ASTCT) criteria [5]. Immune effector cell-associated neurotoxicity syndrome manifested in the index patient with behavioral changes, visual hallucinations, word-finding difficulties, aphasia, apraxia, dizziness, focal to bilateral tonic-clonic seizures, and focal onset seizures with a frequency of approximately 100 per day. This seizure activity could only be stopped after increasing levetiracetam, adding valproic acid, benzodiazepines, and the interleukin (IL) 1 blocker anakinra, which is also effective in CRS [6]. Since the effect of anakinra is not expected until weeks after application [7], the seizures stopped either spontaneously or as a result of the ASD treatment.

The reason why the patient developed ICANS remains speculative because CAR T-cell therapy was already used 14 months before the onset of ICANS, and the pathophysiology of ICANS is not yet fully understood. At least there is evidence that this could be due to the excessive release of cytokines from T lymphocytes [8, 9]. In this context, the IL-6-mediated release of cytokines is thought to lead to endothelial dysfunction, including disruption of the blood-brain barrier (BBB). A dysfunctional BBB leads to the migration of IL-1, IL-6, and IL-15, myeloid cells, and CAR-T cells into the brain [8, 9]. Because IL-1 is released from myeloid cells, it is speculated that IL-1 blocking (e.g., by anakinra) may be beneficial. A second pathophysiological explanation of ICANS could be the evidence from RNA-Seq data that cerebral pericytes express low levels of CD19, which could explain the neurotoxicity of anti-CD19 therapies [8,9]. Furthermore, recent neuropathological studies demonstrated a pattern of multifocal demyelinating leukoencephalopathy associated with a clinical course of severe ICANS [10]. A targeted analysis of glial subtypes also suggested that area-specific cell loss of the oligodendrocyte lineage is a potential cellular and pathophysiologic correlate in severe ICANS [10]. Why the index patient did not develop ICANS earlier than the ninth cycle of glofitamab remains unknown, but it can be speculated that due to a cumulative effect, side effects did not occur until sufficient toxic substrate had accumulated.

There are several arguments why ICANS in the index patient could be due to the administration of glofitamab. First, alternative epilepsy triggers such as genetic epilepsy, infectious, metabolic, endocrine disease, or relapses of lymphoma have been largely ruled out. There was no acute fever, electrolyte disturbance, carcinomatosis, brain metastasis, head trauma, illicit drug dependence, or insomnia that could explain the occurrence of seizures. Second, ICANS has already been reported in association with CD20 blockers [2,11]. Tanaka et al. reported the case of a 55-year-old male with lung cancer who was treated with cisplatin, pemetrexed, nivolumab, and ipilimumab and developed ICANS 100 days after starting therapy [2]. The patient suffered from high fever, disorientation, and seizures and responded positively to glucocorticoids and immunosuppression [2]. Lipe et al. reported the case of a 77-year-old male who developed ICANS after the second dose of talquetamab for relapsed refractory multiple myeloma [11]. After the infusion of siltuximab, the patient recovered completely [11]. Third, due to the long latency period of 14 months between the administration of CAR-T cell therapy and the occurrence of ICANS, it is unlikely that CAR T-cell therapy caused ICANS. Immune effector cell-associated neurotoxicity syndrome following CAR T-cell therapy usually develops a few days or weeks after this therapy [12,13]. In addition, CAR T-cell therapy was tolerated for months without side effects. Fourth, the clinical presentation of the index patient fits the spectrum of adverse effects previously reported with glofitamab. In addition to seizures, he suffered from behavioral changes, hallucinations, aphasia, apraxia, and somnolence. Fifth, the patient had received the last dose of glofitamab one week before the onset of behavioral changes, aphasia, and seizures. Sixth, in a study of 171 patients with B-cell lymphoma who received at least one dose of glofitamab, nine patients experienced transient grade 3 ICANS-like symptoms [14].

Common side effects of glofitamab include swelling of the face, arms, hands, lower legs, or feet, difficulty moving, dizziness or lightheadedness, the feeling of constant movement of oneself or the environment, headache, trembling of hands, sensation of spinning, unsteadiness or clumsiness, arrhythmias, chest pain, dyspnea, cough, muscle weakness in arms, legs, or feet, muscle cramps, muscle pain, muscle stiffness, burning, numbness, tingling, painful sensations pain in hands or feet, swollen joints, joint or bone pain, tender or swollen lymph nodes, rapid weight gain, or swelling at the tumor site [14,15]. Less common side effects of glofitamab include black tarry stools, chest tightness, disorientation, fever or chills, hallucinations, false beliefs that cannot be changed by facts, back or side pain, depression or anxiety, nightmares or unusually vivid dreams, painful or difficult urination, pale skin, sleepiness or unusual drowsiness, sneezing, sore throat, ulcers, sores or white patches in the mouth, unusual bleeding or bruising, or unusual agitation, nervousness or restlessness, unusual tiredness or weakness [3,4,16].

Treatment of ICANS includes glucocorticoids and tocilizumab (a monoclonal antibody that blocks the IL-6 receptor) [7,17]. If ICANS does not respond to glucocorticoids, siltuximab, ruxolitinib, anakinra, dasatinib, and cyclophosphamide can be administered [7]. In pediatric patients with acute lymphoblastic leukemia who developed ICANS after CAR-T-cell therapy, intrathecal hydrocortisone showed a beneficial effect [18]. Recent studies have shown that automated detection of ICANS using quantitative EEG could have predictive value for detecting ICANS and predicting its severity, and therefore could facilitate treatment [19]. As with almost all diseases, early detection of ICANS is necessary in order to improve the outcome [20].

## Conclusions

The case shows that ICANS with drowsiness, behavioral changes, illusions, word-finding difficulties, aphasia, aoraxia, and seizures can develop with glofitamab and that patients with structural cerebral disease may be prone to developing it. Patients receiving glofitamab should undergo cerebral imaging to determine whether previous cerebral lesions may pose an additional risk for developing ICANS. Glofitamab-associated ICANS may develop in the absence of infectious, metabolic, endocrine, cardiovascular, or neoplastic disease. Patients and treating physicians should be made aware of the risk that CD20 blockers may trigger ICANS.
